# Clinical Value of Prognostic Nutritional Index and Neutrophil-to-Lymphocyte Ratio in Prediction of the Development of Sepsis-Induced Kidney Injury

**DOI:** 10.1155/2022/1449758

**Published:** 2022-06-07

**Authors:** Tonghui Xie, Qi Xin, Rui Chen, Xing Zhang, Fengping Zhang, Hong Ren, Chang Liu, Jingyao Zhang

**Affiliations:** ^1^Department of Hepatobiliary Surgery, The First Affiliated Hospital of Xi'an Jiaotong University, Xi'an, Shaanxi 710061, China; ^2^Department of Thoracic Surgery, The First Affiliated Hospital of Xi'an Jiaotong University, Xi'an, Shaanxi 710061, China; ^3^Department of SICU, The First Affiliated Hospital of Xi'an Jiaotong University, Xi'an, Shaanxi 710061, China

## Abstract

**Background:**

Sepsis-related acute kidney injury (S-AKI) is a frequent complication of hospitalized patients and is linked to increased morbidity and mortality. Early prediction and detection remain conducive to optimizing treatment strategies and limiting further insults. This study was aimed at evaluating the potential predictive value of the combined prognostic nutrition index (PNI) and neutrophil-to-lymphocyte ratio (NLR) to predict the risk of AKI in septic patients.

**Methods:**

In this retrospective study, 1238 adult patients with sepsis who were admitted to the First Affiliated Hospital of Xi'an Jiaotong University from January 2015 to June 2021 were enrolled. Patients were divided into two groups: the non-AKI group (*n* = 731) and the S-AKI group (*n* = 507). Univariate and multivariate logistic regression analyses were performed to screen the independent predictive factors of S-AKI. A receiver operating characteristic (ROC) curve was used to evaluate the predictive value of PNI and NLR.

**Results:**

Multivariate logistic regression analysis indicated that age, chronic liver disease, cardiovascular disease, respiratory rate (RR), white blood cells (WBC), blood urea nitrogen (BUN), creatinine (CRE), international normalized ratio (INR), neutrophil-to-lymphocyte ratio (NLR), and prognostic nutrition index (PNI) were independent prognostic factors of S-AKI. In the three models, the adjusted OR of PNI for S-AKI was 0.802 (0.776-0.829), 0.801 (0.775-0.829), and 0.717 (0.666-0.772), while that of NLR was 1.094 (1.078-1.111), 1.097 (1.080-1.114), and 1.044 (1.016-1.072), respectively. In addition, the area under the ROC curve of the PNI plus NLR group was significantly greater than that of the CRE plus BUN group (0.801, 95% CI: 0.775-0.827 vs. 0.750, 95% CI: 0.722-0.778, respectively; *P* < 0.001).

**Conclusions:**

PNI and NLR have been identified as readily available and independent predictors in septic patients with S-AKI. PNI, in combination with NLR, is of vital significance for early warning and efficient intervention of S-AKI and is superior to combined BUN and CRE.

## 1. Introduction

Sepsis is characterized by life-threatening organ dysfunction induced by a dysregulated host response to infection, with a high rate of morbidity and mortality worldwide [1]. The kidneys are highly susceptible to injury in sepsis, and up to 50% of septic patients develop acute kidney injury in intensive care units [2, 3]. Previous studies reported that patients with sepsis-related acute kidney injury (S-AKI) have a mortality rate of approximately 30-45% [4]. In particular, the progression of AKI during sepsis elevated the likelihood of chronic kidney disease as well as other severe organ dysfunction, ultimately resulting in a dramatic public health concern [5]. Therefore, it is imperative to identify effective biomarkers for clinicians for the early diagnosis of septic kidney injury and implement appropriate interventions.

The pathological processes underlying mechanisms of S-AKI are complex and multifaceted. Recent research on S-AKI has indicated that inflammation, immunologic dysregulation, and malnutrition together contribute to disease progression [2, 6, 7]. In sepsis, the invading pathogen binds to pattern recognition receptors (PRR) through pathogen-associated molecular patterns (PAMPs) to initiate immune responses, resulting in the release of large amounts of inflammatory mediators [8]. There can be direct damage to kidney tissue caused by inflammatory mediators that leads to neutrophils infiltrating the renal interstitium. Furthermore, excessive proinflammatory factor release accelerates catabolism, energy and nutritional loss, and the development of fast protein-energy malnutrition (PEM). Inflammatory factors and nutritional indicators may be potential biomarkers to predict the occurrence of S-AKI, while anti-inflammatory and nutritional therapies are vital for the treatment of S-AKI.

Traditionally, serum creatinine and urine output have been employed to diagnose AKI; nonetheless, these indicators are highly susceptible to extrarenal factors and have a significant lag in the detection of renal impairment [9]. In recent years, several novel biomarkers such as neutrophil gelatinase-associated lipocalin (NGAL) [10], urinary tissue inhibitor of metalloproteinase-2 (TIMP-2) [11], soluble thrombomodulin (TM) [12], and kidney injury molecule (KIM-1) [13] have been explored for the diagnosis of AKI. Frustratingly, the majority of those indicators are too costly to apply in clinical practice. The prognostic nutritional index (PNI) and neutrophil-to-lymphocyte ratio (NLR) are composite biomarkers representing patients' immunological nutritional status and systemic inflammation and have the advantages of being simple to use and inexpensive and having improved stability [14, 15]. Previous research has posited that PNI or NLR has prognostic value in numerous illnesses, including sepsis [14], gastric cancer [16], hepatocellular carcinoma [17], and pancreatic cancer [18]. So far, no studies have been conducted to evaluate the predictive value of combined PNI and NLR for S-AKI. Consider that inflammation and immune-nutritional status play a vital role in the physiopathology of S-AKI. Moreover, the sensitivity of a single inflammatory indicator used in clinical practice is inadequate, and additional confounding factors cannot be completely excluded. Therefore, the specific purpose of this study was to systematically elucidate the predictive value of PNI combined with NLR in S-AKI.

## 2. Methods and Materials

### 2.1. Study Design and Population

From January 2015 to June 2021, data on 1238 patients diagnosed with sepsis were retrospectively collected from the First Affiliated Hospital of Xi'an Jiaotong University. The inclusion criteria were as follows: (1) sepsis, defined by the Third International Consensus Definitions as a sequential organ failure assessment score ≥ 2 points and suspected infection [1]; (2) the diagnosis of AKI, defined by the Kidney Disease Improving Global Outcomes clinical practice guideline [19] as an increase in serum creatinine levels of 0.3 mg/dL (26.5 mol/L) within 48 hours, or 1.5 times from baseline within 48 hours within 7 days, or an accumulated 6-hour urine volume of 0.5 mL/kg/h. The exclusion criteria were as follows: (1) patients under the age of 18 years; (2) duration of the hospital stay less than 48 hours; (3) at the time of admission, there was insufficient clinical and laboratory data; (4) hematological disorders; (5) evidence of chronic kidney disease, end-stage renal disease, or a history of kidney transplantation; (6) pregnant or lactating women.

### 2.2. Data Collection

All patient data were collected from the First Affiliated Hospital of Xi'an Jiaotong University's electronic medical record system. The following data were extracted: (1) demographic parameters, including age and gender; (2) vital signs, including body temperature (T), respiratory rate (RR), heart rate (HR), systolic blood pressure (SBP), diastolic blood pressure (DBP), and mean arterial pressure (MAP); (3) comorbidities such as diabetes, hypertension, cardiovascular disease, chronic liver disease, and malignancy; and (4) within 24 hours of admission, laboratory measurement results such as white blood cell (WBC) count, neutrophil count, lymphocyte count, platelet count, monocyte count, red cell distribution width (RDW), albumin (ALB), glucose (GLU), alanine aminotransferase (ALT), aspartate aminotransferase (AST), total bilirubin (TBIL), creatinine (CRE), blood urea nitrogen (BUN), and uric acid (UA) were collected. As reported in previous studies, NLR was defined as the ratio of the absolute neutrophil count to the absolute lymphocyte count [14]; PNI was calculated by the following formula: serum albumin (g/L) + 5 × lymphocyte count (10^9^/L) [20]. PLR was calculated by the absolute platelet count divided by the absolute lymphocyte count [21].

### 2.3. Statistical Analysis

All patients' baseline characteristics and laboratory data were stratified based on whether or not they developed AKI. Variables were classified as either continuous or categorical variables. Shapiro-Wilk tests were used to evaluate variable distributions. Continuous variables following the normal distribution were expressed as mean ± standard deviation, whereas variables not following the normal distribution were represented as median and interquartile ranges. In univariate analyses, Student's *t*-test and the Wilcoxon Mann–Whitney test were used to compare continuous variables between the non-AKI and S-AKI groups. Categorical variables were represented as frequencies with percentages and compared using the chi-square test or Fisher's exact test. Multivariate logistic regression analysis was performed to identify variables associated with incident S-AKI and adjusted for confounding factors to determine the predictive value of the combination of PNI and NLR on the occurrence of S-AKI. Receiver operator characteristic (ROC) curve analysis was used to further analyze the accuracy of various indicators for S-AKI. The *Z* test was used to compare the area under the ROC curve (AUC). All statistical analyses were carried out by SPSS 24.0, and *P* values < 0.05 were considered statistically significant.

## 3. Results

### 3.1. Demographic and Baseline Characteristics

**Figure 1:**
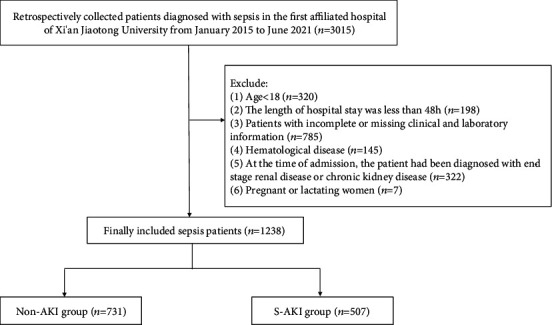
A flow diagram of patient enrollment.

The patient enrollment flow diagram is illustrated in Figure 1. A total of 1238 patients with sepsis were enrolled in the final analysis. According to the study's objectives, patients were then divided into two groups, and 507 patients were eventually clinically diagnosed with AKI. The median age of patients was 58, with ages ranging from 46 to 68. Female patients accounted for 36.19% of the total sample. The demographic and clinical characteristics of patients in the non-AKI and the S-AKI groups are presented in Table 1. Patients with S-AKI were significantly older than those without AKI (60, 49-70 vs. 56, 45-66; *P* < 0.001). Moreover, there were significant differences between patients in the non-AKI group and S-AKI group in RR (20, 18-22 vs. 21, 19-24, respectively; *P* < 0.001), DBP (75, 65-79 vs. 70, 64-80, respectively; *P* < 0.001), MAP (88, 79-94 vs. 83, 76-95, respectively; *P* < 0.001), and comorbidities such as diabetes (18.88 vs. 25.64%, respectively; *P* = 0.001), hypertension (18.60 vs. 29.59%, respectively; *P* < 0.001), cardiovascular disease (10.40 vs. 20.91%, respectively; *P* < 0.001), and chronic liver disease (20.52 vs. 29.39%, respectively; *P* < 0.001). Meanwhile, no statistically significant differences in HR, temperature, SBP, and malignancy were noted between the two groups (*P* > 0.05).

### 3.2. Univariate Analyses of Clinical and Laboratory Results in Patients with Sepsis within the First 24 h

Clinical and laboratory results during and 24 h following admission are listed in Table 2. Univariate analyses revealed a significant difference between the non-AKI and S-AKI groups in terms of WBC count (9.15, 6.06-13.31 vs. 11.23, 7.03-16.32, respectively; *P* < 0.001), neutrophil count (6.71, 4.12-9.70 vs. 10.02, 6.69-14.62, respectively; *P* < 0.001), lymphocyte count (0.93, 0.64-1.24 vs. 0.67, 0.44-0.95, respectively; *P* < 0.001), platelet count (167.00, 103.00-236.50 vs. 141.00, 85.00-210.00, respectively; *P* < 0.001), ALB (32.60, 29.50-35.90 vs. 27.80, 25.10-31.30, respectively; *P* < 0.001), AST (31.00, 20.00-56.00 vs. 42.00, 24.00-106.00, respectively; *P* < 0.001), ALT (30.00, 18.00-56.00 vs. 40.00, 20.00-89.00, respectively; *P* < 0.001), TBIL (21.20, 12.70-43.00 vs. 26.70, 14.30-70.70, respectively; *P* < 0.001), CRE (56.00, 44.00-77.00 vs. 101.00, 60.00-186.00, respectively; *P* < 0.001), BUN (5.91, 4.18-9.24 vs. 11.77, 6.20-18.40, respectively; *P* < 0.001), UA (232.00, 158.00-312.00 vs. 316.00, 217.00-443.00, respectively; *P* < 0.001), INR (1.22, 1.11-1.38 vs. 1.30, 1.13-1.59, respectively; *P* < 0.001), NLR (7.38, 4.22-12.41 vs. 14.86, 9.58-24.89, respectively; *P* < 0.001), PNI (37.00, 34.70-39.85 vs. 31.70, 31.10-35.85, respectively; *P* < 0.001), and PLR (190.24, 108.98-297.01 vs. 213.79, 114.71-355.56, respectively; *P* = 0.013). However, these differences were not significant for monocyte count, RDW, and GLU.

### 3.3. Multivariate Logistic Regression Analysis of Risk Factors for S-AKI in Septic Patients

Multivariable binary logistic regression analysis was used to identify potential predictors of S-AKI in septic patients. Variables included age, sex, diabetes, RR, MAP, WBC count, platelet count, AST, ALT, TBIL, BUN, CRE, INR, UA, PNI, PLR, and NLR, as well as diabetes, hypertension, cardiovascular disease, chronic liver disease, and malignancy. As outlined in Table 3, age (OR = 1.016; 95% CI: 1.005-1.027), RR (OR = 1.130; 95% CI: 1.084-1.178), WBC (OR = 1.029; 95% CI: 1.008-1.050), BUN (OR = 1.062; 95% CI: 1.027-1.098), CRE (OR = 1.008; 95% CI: 1.005-1.012), INR (OR = 1.410; 95% CI: 1.103-1.803), PNI (OR = 0.841; 95% CI: 0.810-0.873), and NLR (OR = 1.070; 95% CI: 1.053-1.088), as well as chronic liver disease (OR = 1.775; 95% CI: 1.248-2.523) and cardiovascular disease (OR = 1.986; 95% CI: 1.283-3.073), were independent predictors of S-AKI in septic patients. The adjusted OR of PNI for S-AKI was 0.802 (95% CI: 0.776-0.829), 0.801 (95% CI: 0.775-0.829), and 0.717 (95% CI: 0.666-0.772) in the three models, while the adjusted OR of NLR for S-AKI was 1.094 (95% CI: 1.078-1.111), 1.097 (95% CI: 1.080-1.114), and 1.044 (95% CI: 1.016-1.072), respectively (Table 4).

### 3.4. Predictive Value of PNI and NLR for Patients with S-AKI

**Figure 2:**
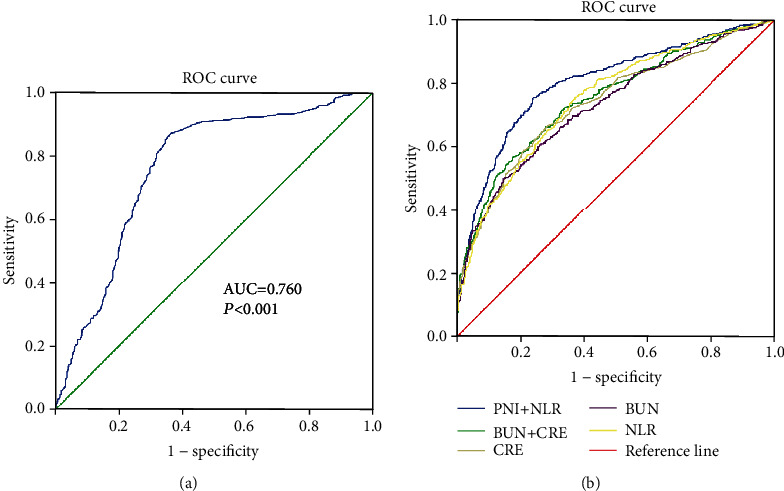
Receiver operating characteristic curve analyses of independent predictors for S-AKI. The ROC curve of PNI in predicting sepsis patients with S-AKI (a). The ROC curve of NLR, BUN, CRE, PNI+NLR, and BUN+CRE in predicting sepsis patients with S-AKI (b). PNI: prognostic nutrition index; NLR: neutrophil-to-lymphocyte ratio; CRE: creatinine; BUN: blood urea nitrogen.

According to ROC curve analysis, the most influential indicators for patients with S-AKI were PNI (AUC 0.760; 95% CI: 0.731-0.789, *P* < 0.001) and NLR (AUC = 0.749; 95% CI: 0.722-0.777, *P* < 0.001), as depicted in Table 5 and Figure 2. Then, the predictive values of combined PNI+NLR and BUN+CRE were also compared in septic patients with S-AKI. It was uncovered that the combination of PNI+NLR had a higher predictive value for patients with S-AKI than combined BUN+CRE, with a statistically significant difference in AUC (0.801 vs. 0.750; *P* = 0.003).

## 4. Discussion

In this retrospective study of 1238 patients with sepsis in our hospital, the clinical predictive value of PNI combined with NLR was systematically investigated for S-AKI. In septic patients, a higher NLR and lower PNI on admission were significantly correlated with an increased risk of S-AKI. In the present study, NLR and PNI could be used as independent predictors of S-AKI. In addition, to our knowledge, this is the first study to explore the effect of NLR in combination with PNI in sepsis-induced kidney damage.

Acute kidney injury, defined as a rapid decrease in glomerular filtration rate that results in raised serum creatinine levels, imposes a serious disease burden on hospitalized patients. Sepsis is the leading cause of AKI, especially in individuals with severe sepsis [2, 22]. Early sepsis is characterized by an organism's proinflammatory response mediated by neutrophils, macrophages, and other immune cells, referred to as the hyperdynamic phase. The organism subsequently enters a hypokinetic period marked by decreased tissue perfusion, impaired microcirculation, and exacerbated organ damage [23]. Meanwhile, sepsis-induced tissue damage increases inducible nitric oxide (NO) synthase activity while substantially suppressing endothelial NO synthase activity. Endothelial-dependent vasodilation mediated by nitric oxide is indeed minimized by NO synthase dysfunction, which inevitably results in local renal microcirculation imbalance [24]. It is worth mentioning that inflammatory cell infiltration plays a crucial role in the development of S-AKI [25]. As highlighted in our study, WBC (11.23, 7.03-16.32 vs. 9.15, 6.06-13.32) and neutrophil (10.02, 6.69-14.62 vs. 6.71, 4.12-9.70) counts were considerably higher in the S-AKI group than in the non-AKI group. Furthermore, our study uncovered that advanced age, chronic liver disease, and cardiovascular disease were independent predictors of acute renal injury in sepsis, which is consistent with previous research [3, 26]. With increasing age, the body's self-regulatory mechanism is perturbed, and the secretion of vasoactive substances decreases, resulting in a gradual decrease in the number of functional glomeruli, which eventually expedites the occurrence and progression of acute kidney injury. We included septic patients with chronic liver disease and complications such as abdominal effusion and lower extremity edema that resulted in decreased effective circulating blood volume, activation of the sympathetic adrenal medulla system, elevated catecholamine concentrations in the blood, and significantly decreased glomerular filtration rate, which eventually induced renal damage [27, 28].

PNI was developed to assess the preoperative nutritional immune status and postoperative complications in surgical patients and has since been demonstrated to be a reliable prognostic factor for numerous malignancies, such as gastric cancer [16], intrahepatic cholangiocarcinoma [29], esophageal cancer [30], and nasopharyngeal carcinoma [31]. In the field of sepsis, Wu et al. reported that after adjusting for confounding variables, PNI was asserted to be an independent prognostic factor for all-cause mortality at 30 days [32]. Additional studies have concluded that the presence and severity of neonatal sepsis were negatively and independently associated with PNI. However, studies concentrating on the role of PNI in predicting the development of AKI in septic patients are limited. These patients have an excessive depletion of albumin as a result of a large number of inflammatory factors in the body, leading to a negative nitrogen balance, tissue hypoxia, and impaired microcirculation, thereby resulting in increased capillary permeability and a further decrease in albumin level. A previous study demonstrated that low albumin level was an effective predictor of mortality in ICU septic patients [33]. Our study determined that serum albumin (27.8, 25.10-31.30 vs. 32.60, 29.50-35.90) was significantly lower in S-AKI patients than in non-AKI patients. In addition to nutritional assessment, immunosuppression plays a pivotal role in the pathogenesis of sepsis. In the context of sepsis, pathogens evade the body's immune system's protective mechanisms and persist in proliferating and secreting a large number of inflammatory mediators that aggravate host cell damage or death, eventually leading to immune system imbalance [34, 35]. Some of these immune cells (CD4+ T cells, B cells, follicular dendritic cells, etc.) suffer from the impact of apoptosis, which was especially noticeable in the immunosuppressive link. Various complications, particularly compromised renal function, were caused by a significant decrease in the number of immune cells in tissues and organs as well as a progressive decline in immune function. Our research demonstrated that PNI (OR = 0.841; 95% CI: 0.810-0.873) was an independent predictor of S-AKI in septic patients. Indeed, the sensitivity and specificity were 86.9% and 63.7%, respectively. Moreover, the adjusted OR of PNI for S-AKI was 0.802 (0.776-0.829), 0.801 (0.775-0.829), and 0.717 (0.666-0.772) in the three models, respectively.

NLR is a well-established indicator of systemic inflammatory response that plays a key role in the diagnosis and prognosis of sepsis [14, 36]. On the one hand, infection by pathogens stimulates neutrophils to secrete proinflammatory cytokines, regulatory cytokines, and chemokines, leading to varying degrees of organ damage. On the other hand, lymphocytes suppress the body's inflammatory response by secreting anti-inflammatory factors such as interleukin-10 (IL-10). The two mentioned above keep the immune system in balance [37]. Studies have established that when the body experiences an overwhelming inflammatory response, such as sepsis, the number of lymphocytes is drastically reduced, resulting in an immunosuppressive state [38]. In clinical practice, NLR is a common, easily accessible serological indicator that effectively responds to fluctuations in the condition of septic patients. Besides, studies have connected the initial NLR measured at admission to 28-day mortality [39]. An increased level of NLR was apparently associated with the progression of S-AKI in septic patients and could be regarded as risk stratification of S-AKI [40]. Similarly, we also discovered that NLR was an independent predictor of S-AKI in septic patients, with a sensitivity of 75.3% and a specificity of 63.1%. Additionally, the adjusted OR of NLR for S-AKI was 1.094 (1.078-1.111), 1.097 (1.080-1.114), and 1.044 (1.016-1.072), respectively.

Inflammation, immunity, and nutrition all play a critical role in the pathogenesis and progression of sepsis. Septic patients produce a high level of inflammatory mediators and catabolic hormones, which facilitate catabolic metabolism and weaken anabolic metabolism, resulting in severe malnutrition, immunosuppression, and an escalating inflammatory response. In this study, compared with the combination of traditional renal injury monitoring indicators BUN+CRE (AUC = 0.750), the combination of PNI and NLR was able to greatly enhance the accuracy in predicting the occurrence of S-AKI (AUC = 0.801). Among them, the sensitivity of PNI+NLR was significantly higher than that of BUN+CRE (75.3 vs. 56.6, respectively). It is worthwhile pointing out that in investigating the course of renal injury in diverse septic patients more comprehensively, patients with cirrhosis, malignancy, and other underlying adverse conditions were also included. That might also be one of the reasons underlying the lower specificity of PNI+NLR compared to BUN+CRE (76.1 vs. 82.5, respectively).

Nevertheless, our study has several limitations, which are summarized as follows: (1) This was a single-center retrospective study with a small sample size. As a result, prospective follow-up studies with a large sample size are necessitated to validate our findings and confirm the predictive efficiency of combined PNI and NLR. (2) Although we investigated the risk factors for AKI in hospitalized septic patients, it is conceivable that not all of them were considered. (3) We only included serological indicators within the first 24 hours of admission and did not dynamically analyze alterations in patient indicators during hospitalization. (4) Due to a lack of corresponding follow-up data, we were unable to confirm the impact of risk factors on subsequent patient survival status. (5) Owing to missing data, other inflammatory biomarkers like C-reactive protein and lactate were not assessed. (6) The retrospective study had a lengthy data collection period, and advances in septic treatment technologies during this time period may have influenced clinical outcomes.

## 5. Conclusions

The assessment of prognostic risk in patients with sepsis has always been challenging in clinics. Our study indicated that RR, WBC, BUN, CRE, INR, NLR, PNI, and chronic liver and cardiovascular diseases were independent predictors of S-AKI in septic patients. Furthermore, the combination of the inflammatory marker NLR and the immune-nutritional marker PNI has a higher diagnostic value than the traditional markers CRE and BUN, signaling that they may have complementary advantages and improve the accuracy of early prediction of S-AKI.

## Figures and Tables

**Table 1:** Demographic characteristics between non-AKI group and S-AKI group.

Variables	Total	Non-AKI	S-AKI	*P* value
	*n* = 1238	n = 731	*n* = 507	
Age (years)	58 (46-68)	56 (45-66)	60 (49-70)	<0.001
Female, *n* (%)	448 (36.19)	276 (37.77)	172 (33.93)	0.168
Vital signs				
HR (bpm)	93 (80-109)	92 (79-110)	95 (80-109)	0.262
RR (bpm)	20 (19-22)	20 (18-22)	21 (19-24)	<0.001
T (°C)	36.6 (36.3-37.1)	36.6 (36.3-37.0)	36.6 (36.3-37.2)	0.321
SBP (mmHg)	114 (102-127)	115 (103-126)	112 (101-128)	0.229
DBP (mmHg)	72 (65-79)	75 (65-79)	70 (64-80)	0.032
MAP (mmHg)	86 (78-94)	88 (79-94)	83 (76-95)	0.033
Comorbidities, *n* (%)				
Diabetes	268 (21.65)	138 (18.88)	130 (25.64)	0.004
Hypertension	286 (23.10)	136 (18.60)	150 (29.59)	<0.001
Cardiovascular disease	182 (14.70)	76 (10.40)	106 (20.91)	<0.001
Chronic liver disease	299 (24.15)	150 (20.52)	149 (29.39)	<0.001
Malignancy tumor	292 (23.59)	169 (23.12)	123 (24.26)	0.642

HR: heart rate; RR: respiratory rate; T: temperature; SBP: systolic blood pressure; DBP: diastolic blood pressure; MAP: mean arterial pressure.

**Table 2:** Univariate analyses of clinical and laboratory results between non-AKI group and S-AKI group within the first 24 h data.

Variables	Total	Non-AKI	S-AKI	*P* value
	*n* = 1238	*n* = 731	*n* = 507	
WBC (10^9^/L)	9.76 (6.47-14.73)	9.15 (6.06-13.32)	11.23 (7.03-16.32)	<0.001
Neutrophil (10^9^/L)	8.16 (4.78-11.85)	6.71 (4.12-9.70)	10.02 (6.69-14.62)	<0.001
Lymphocyte (10^9^/L)	0.81 (0.54-1.13)	0.93 (0.64-1.24)	0.67 (0.44-0.95)	<0.001
Platelet (10^9^/L)	156.00 (99.00-228.00)	167.00 (103.00-236.50)	141.00 (85.00-210.00)	<0.001
Monocyte (10^9^/L)	0.41 (0.25-0.65)	0.40 (0.25-0.62)	0.43 (0.25-0.68)	0.090
RDW (%)	14.00 (13.00-15.60)	13.90 (12.90-15.50)	14.10 (13.10-15.70)	0.129
ALB (g/L)	31.00 (27.40-34.43)	32.60 (29.50-35.90)	27.80 (25.10-31.30)	<0.001
GLU (mmol/L)	6.72 (5.19-9.68)	6.66 (5.21-9.63)	6.95 (5.10-9.82)	0.650
ALT (U/L)	33.00 (20.00-69.00)	30.00 (18.00-56.00)	40.00 (22.00-89.00)	<0.001
AST (U/L)	35.00 (21.00-73.00)	31.00 (20.00-56.00)	42.00 (24.00-106.00)	<0.001
TBIL (*μ*mol/L)	23.45 (13.10-56.00)	21.20 (12.70-43.00)	26.70 (14.30-70.70)	<0.001
CRE (*μ*mol/L)	65.00 (4800-114.25)	56.00 (44.00-77.00)	101.00 (60.00-186.00)	<0.001
BUN (mmol/L)	7.21 (4.73-13.21)	5.91 (4.18-9.24)	11.77 (6.20-18.40)	<0.001
UA (*μ*mol/L)	262.00 (178.75-365.00)	232.00 (158.00-312.00)	316.00 (217.00-443.00)	<0.001
INR	1.25 (1.11-1.46)	1.22 (1.11-1.38)	1.30 (1.13-1.59)	<0.001
NLR	10.07 (5.40-16.87)	7.38 (4.22-12.41)	14.86 (9.58-24.89)	<0.001
PNI	35.55 (31.65-38.80)	37.00 (34.70-39.85)	31.70 (31.10-35.85)	<0.001
PLR	200.00 (110.51-317.88)	190.24 (108.98-297.01)	213.79 (114.71-355.56)	0.013

WBC: white blood cell; RDW: red cell distribution width; ALB: albumin; GLU: glucose; ALT: alanine aminotransferase; AST: aspartate aminotransferase; TBIL: total bilirubin; CRE: creatinine; BUN: blood urea nitrogen; UA: uric acid; INR: international normalized ratio; NLR: neutrophil-to-lymphocyte ratio; PNI: prognostic nutrition index; PLR: platelet-to-lymphocyte ratio.

**Table 3:** Multivariate logistic regression analysis of risk factors for S-AKI in septic patients.

Variable	OR	95% CI	*P* value
Age	1.016	1.005-1.027	0.005
Chronic liver disease	1.775	1.248-2.523	0.001
Cardiovascular disease	1.986	1.283-3.073	0.002
RR	1.130	1.084-1.178	<0.001
WBC	1.029	1.008-1.050	0.006
BUN	1.062	1.027-1.098	<0.001
CRE	1.008	1.005-1.012	<0.001
INR	1.410	1.103-1.803	0.006
NLR	1.070	1.053-1.088	<0.001
PNI	0.841	0.810-0.873	<0.001

OR: odds ratio; CI: confidence interval; RR: respiratory rate; WBC: white blood cell; BUN: blood urea nitrogen; CRE: creatinine; INR: international normalized ratio; NLR: neutrophil-to-lymphocyte ratio; PNI: prognostic nutrition index.

**Table 4:** Association of PNI and NLR with AKI in sepsis patients.

Exposure	Nonadjusted OR	*P* value	Adjusted OR	*P* value
*Model 1*				
PNI	0.799 (0.773-0.825)	<0.001	0.802 (0.776-0.829)	<0.001
NLR	1.094 (1.077-1.110)	<0.001	1.094 (1.078-1.111)	<0.001
*Model 2*				
PNI	0.799 (0.773-0.825)	<0.001	0.801 (0.775-0.829)	<0.001
NLR	1.094 (1.077-1.110)	<0.001	1.097 (1.080-1.114)	<0.001
*Model 3*				
PNI	0.799 (0.773-0.825)	<0.001	0.717 (0.666-0.772)	<0.001
NLR	1.094 (1.077-1.110)	<0.001	1.044 (1.016-1.072)	0.002

Model 1: adjusted for age and sex. Model 2: model 1 and diabetes, hypertension, cardiovascular disease, chronic liver disease, malignancy tumor, HR, RR, and MAP. Model 3: model 2 and WBC, neutrophil, lymphocyte, platelet, monocyte, RDW, ALB, GLU, AST, ALT, CRE, BUN, UA, and INR. HR: heart rate; RR: respiratory rate; MAP: mean arterial pressure; RDW: red cell distribution width; ALB: albumin; GLU: glucose; ALT: alanine aminotransferase; AST: aspartate aminotransferase; CRE: creatinine; BUN: blood urea nitrogen; UA: uric acid; INR: international normalized ratio.

**Table 5:** Receiver operating curve (ROC) for prediction in S-AKI patients.

Indicator	AUC	95% CI	*P*	Optimal cut-off value	Sensitivity (%)	Specificity (%)
PNI	0.760	0.731-0.789	<0.001	32.75	86.9	63.7
NLR	0.749	0.722-0.777	<0.001	9.54	75.3	63.1
CRE	0.737	0.709-0.766	<0.001	72.5	65.9	72.1
BUN	0.729	0.700-0.757	<0.001	11.82	49.9	85.5
PNI+NLR	0.801	0.775-0.827	<0.001	—	75.3	76.1
BUN+CRE	0.750	0.722-0.778	<0.001	—	56.6	82.5

PNI: prognostic nutrition index; NLR: neutrophil-to-lymphocyte ratio; CRE: creatinine; BUN: blood urea nitrogen.

## Data Availability

The datasets used and/or analysed during the current study are available from the corresponding author on reasonable request.
